# *Coreopsis tinctoria* and Its Flavonoids Ameliorate Hyperglycemia in Obese Mice Induced by High-Fat Diet

**DOI:** 10.3390/nu14061160

**Published:** 2022-03-09

**Authors:** Feng Zhang, Minglan Yang, Jia Xu, Yanzhou Hu, Ruxin Gao, Kunlun Huang, Xiaoyun He

**Affiliations:** 1 Key Laboratory of Precision Nutrition and Food Quality, Ministry of Education, College of Food Science and Nutritional Engineering, China Agricultural University, Beijing 100083, China; zhangfengyx818929@163.com (F.Z.); yangminglan@aliyun.com (M.Y.); xujia1995012@126.com (J.X.); huyz369@163.com (Y.H.); ruxin1958@163.com (R.G.); hkl009@163.com (K.H.); 2 Department of Clinical Nutrition, West China Hospital, Sichuan University, Chengdu 610041, China; 3 Key Laboratory of Safety Assessment of Genetically Modified Organism (Food Safety), The Ministry of Agriculture and Rural Affairs of the P.R. China, Beijing 100083, China

**Keywords:** obesity, *Coreopsis tinctoria*, kaempferol, glucose tolerance, insulin resistance, gut microbiota

## Abstract

With the prevalence of obesity all over the world, human health has been seriously affected. In particular, the number of diabetic and cardiovascular diseases has increased dramatically. The herb *Coreopsis tinctoria* (*C. tinctoria*) shows diverse biological and pharmacological activities, which are mainly attributed to its flavonoids. However, the specific functional substances that play an active role in *C. tinctoria* remain unclear, and its mechanism has not been deeply explored. In this study, we established a diet-induced obesity (DIO) mice model and treated mice with *C. tinctoria* or kaempferol for 8 weeks. The results showed that both *C. tinctoria* and kaempferol lowered body weight, reduced fasting blood glucose, and improved glucose tolerance and insulin resistance to alleviate obesity in DIO mice. The level of hemoglobin A1c also decreased significantly after treatment with *C. tinctoria* and kaempferol. Moreover, the administration of *C. tinctoria* and kaempferol also restored gut microbiota imbalance and significantly increased *Desulfovibrio* and *Butyricimonas* levels, which have been reported to improve glucose metabolism and intestinal health. In general, our study shows that *C. tinctoria* is a potential hypoglycemic substance for obesity and may reduce blood glucose by regulating gut microbiota, and that kaempferol is one of the effective substances of *C. tinctoria*.

## 1. Introduction

In recent years, rates of global obesity have increased dramatically. According to World Health Organization (WHO) estimations, one in five adults will be obese by 2025 [1]. In fact, obesity has been almost considered as an epidemic worldwide, associated with diabetes mellitus, cardiovascular disease, fatty liver disease, osteoarthritis, obstructive sleep apnea, several cancers, as well as social and psychological problems [2,3]. Overweight and lack of physical activity are the main determinants of insulin resistance with hyperglycemia and hyperinsulinemia. Furthermore, reports indicate that severe obesity in childhood and adolescence increases the risk of type 2 diabetes (T2DM) in youth and young adults [4,5]. There is sufficient evidence that obesity management can delay the development of T2DM from pre-diabetes and may be beneficial to its treatment [6]. At present, most obesity or diabetes treatment methods have the disadvantages of significant side effects, easy rebound, decreased immunity, and so on [7,8]. Therefore, looking for a safe and effective way to lose weight and reduce blood glucose has become the consistent direction of public health efforts. In recent years, dietary intervention has become a main research hotspot [9].

*Coreopsis tinctoria* (*C. tinctoria*) is an annual herb that mainly grows in high-altitude areas above 3000 m in Xinjiang, China, especially the Hotan area [10]. Current studies have shown that its water extracts possess diverse biological and pharmacological activities, among them anti-diabetes, anti-cardiovascular diseases, antioxidant, and protective effects on organs [11,12,13,14]. *C. tinctoria* flower tea ameliorated hepatic steatosis, glucose intolerance, and insulin resistance in HFD rats, and the regulatory effects may be mediated via the Akt/FoxO1 signaling pathway [15]. A study showed that *C. tinctoria* tea administration could significantly increase the intestinal microbial richness and diversity of mice induced by a high-fat diet and improve gut microbiota composition to an approximately normal status, thereby regulating blood lipid metabolism [14]. The major bioactive components in *C. tinctoria* are the flavonoids [16,17]. Previous studies have found that the flavonoids from *C. tinctoria* extracts can reduce blood lipid by down-regulating adipose differentiation-related protein ADRP [18]. Kaempferol is a major flavonoid glycoside found in many natural products, also found in *C. tinctoria*, which displays several pharmacological properties, including anti-inflammatory, antioxidant, antitumor, neuroprotective, and antidiabetic activities [19,20]. It is not clear whether kaempferol is one of the hypoglycemic constituents of *C. tinctoria*.

In this study, we explored the hypoglycemic effects of water extract of *C. tinctoria* and kaempferol on a hyperglycemia model induced by a high-fat diet, and investigated whether the modulation of gut microbiota is the mechanism for *C. tinctoria* extracts and kaempferol to exert their effects on the hyperglycemia model.

## 2. Materials and Methods

### 2.1. Preparation of Water Extract of C. tinctoria and Single Flavonoids Substance

*C. tinctoria* was collected from the planting base 5500 m above sea level in Saitula, Xinjiang, China. The petals of the plants were crushed and sieved with a 60 mesh sieve with 0.3 mm square holes. Afterwards, *C. tinctoria* was collected using water as an extractant. *C. tinctoria* was extracted by rotating and evaporating at 80 °C for 45 min according to the ratio of material to liquid of 1:10, and the process was repeated three times. The water extract was then freeze-dried for 48 h to obtain *C. tinctoria*. The components of water extract in *C. tinctoria* were determined by liquid chromatography mass spectrometry (LC-MS) with TripleTOF5600^+^ (AB SCIEX™, Boston, MA, USA). Kaempferol (Purity ≥ 95%, HPLC grade) was purchased from Dalian Meilun Biotechnology Co., Ltd. (Dalian, China).

### 2.2. Animals and Experimental Design

This study was performed in accordance with protocols approved by the Animal Ethics Committee of Agricultural Product Quality Supervision, Inspection, and Testing Center (Beijing) of Ministry of Agriculture, China Agricultural University (KY19037, Beijing, China). Four-week-old male C57BL/6J mice (12–15 g) were purchased from Vital River Laboratory Technology Co., Ltd. (Beijing, China). Animals received sterile water and food ad libitum and were housed in standard plastic cages under a 12 h light/dark cycle at a controlled temperature (23 ± 2 °C). The 45% high-fat diet used in this experiment was purchased from Beijing HFK Bioscience Co., Ltd. (Beijing, China), and the normal diet was purchased from Beijing KEAO XIELI Feed Co., Ltd. (Beijing, China). Prior to the experiment, the mice were acclimated to the new environment for 1 week. Then, the diet-induced obesity (DIO) mice models (n = 32) were established by feeding the high-fat diet and the control check (CK, n = 8), which allowed access to the normal diet, until there were significant differences in body weight and blood glucose between DIO mice and CK mice at 10 weeks. After that, the DIO mice (n = 32) were randomly divided into four groups: the high-fat diet group (HFD, n = 8), the HFD+Kaempferol-Low group (KaeL, n = 8), the HFD+Kaempferol-High group (KaeH, n = 8), and the HFD+water extract of *C. tinctoria* group (Ct, n = 8). The KaeL group and KaeH group were administered kaempferol at concentrations of 25 mg/kg or 50 mg/kg (0.1 mL/10 g bodyweight) daily by oral gavage. Mice in the Ct group were treated with 0.4 g/mL water extract of *C. tinctoria* (0.1 mL/10 g bodyweight). CK and HFD groups were administered sterile water (0.1 mL/10 g bodyweight) daily by oral gavage. The mice were treated for eight weeks. During the treatment period, the animal behavior was observed daily, and body weight was measured weekly. Fasting blood glucose was measured at the 10th week, at the 12th week, at the 15th week, and at the 18th week. At the 18th week, mouse fecal samples were collected. Then, mice were anesthetized and euthanized by exsanguination, and blood samples and tissues were collected.

### 2.3. ITT

At the 16th week, mice were fasted for 4 h, and then an intraperitoneal insulin tolerance test (ITT) was performed after insulin injection (0.70 U per kg body weight). The tail blood was collected at 0 min, 15 min, 30 min, 45 min, and 60 min, and blood glucose was measured. The AUC for ITT was calculated by the trapezoidal rule.

### 2.4. GTT

At the 17th week, GTT was performed. Mice were fasted for 16 h, and then intraperitoneally (i.p.) injected with 1.5 g/kg body weight glucose. The blood glucose values of each mouse were measured at 0 min, 15 min, 30 min, 60 min, 90 min, and 120 min. The area under the curve (AUC) was given as the incremental AUC calculated by the conventional trapezoid rule.

### 2.5. Biochemical Analysis

High-density lipoprotein (HDL), low-density lipoprotein (LDL), triacylglycerols (TG), total cholesterol (TC), alkaline phosphatase (ALP), aspartate transaminase (AST), alanine aminotransferase (ALT), total protein (TP), albumin (ALB), uric acid (UA), creatinine (CREA), blood urea nitrogen (BUN) and glucose (GLU) in serum were determined using an automatic biochemical analyzer (Hitachi, 7600-020, Tokyo, Japan). Free fat acid (FFA) in the serum was measured with the kits of enzyme-linked immunosorbent assay according to the manufacturer’s recommendations (Nanjing Jiancheng Bioengineering Institute, Nanjing, China).

### 2.6. Hemoglobin A1c Analysis

In the last week of the experiment, mice were sacrificed and hemoglobin a1c (HbA1c) was measured in whole blood with glycosylated hemoglobin enzyme-linked immunosorbent assay kits (Beijing Sinouk Institute of Biological Technology, Beijing, China).

### 2.7. Gut Microbiota Analysis

Fresh mouse fecal samples were collected from all groups and immediately frozen at −80 °C in the last week. Fecal microbial genomic DNA was extracted using the Stool Genomic DNA Extraction Kit (Beijing Solarbio Technology Co., Ltd., Beijing, China) following the manufacturer’s protocol. Then, the V3–V4 hypervariable regions of 16S ribosomal DNA (rDNA) were amplified with High-Fidelity Polymerase and Phusion^®^ High-Fidelity PCR Master Mix with GC Buffer using specific primers with barcode. Sequencing libraries were established by the TruSeq^®^ DNA PCR-Free Sample Preparation Kit, and all samples were sequenced on the HiSeq2500 PE250 platform (Illumina, San Diego, CA, USA). Further processing of raw sequencing data was performed according to the Qiime quality control procedure (V1.7.0, http://qiime.org/scripts/split_libraries_fastq.html (accessed on 3 January 2020)). The lowest amount of data in the sample is used as the standard for normalization. Gut microbiota data analyses were performed by PAST3 software, Excel, Heatmap Illustrator1.0, and Huttenhower Lab Galaxy Server.

### 2.8. Statistical Analysis

Experimental data are presented as mean ± standard deviation (SD). The significant differences between groups were determined by one-way ANOVA with Tukey’s post hoc test. A difference was considered to be significant at *p* < 0.05. *p* values of less than 0.05, 0.01, 0.001, and 0.0001 are expressed as * *p* < 0.05 (vs. HFD group), ** *p* < 0.01 (vs. HFD group), *** *p* < 0.001, and **** *p* < 0.0001 (vs. HFD group), respectively. Graphing of the data was carried out using Graph Prism.

## 3. Results

### 3.1. Components of the Extract of C. tinctoria

As shown in Table 1, flavonoids are the main components of water extract in *C. tinctoria*, among them kaempferol, eriodictyol, luteolin, and quercetin-3-O-glucoside.

### 3.2. C. tinctoria and Kaempferol Reduced Body Weight and Improved Obesity in DIO Mice

**Figure 1:**
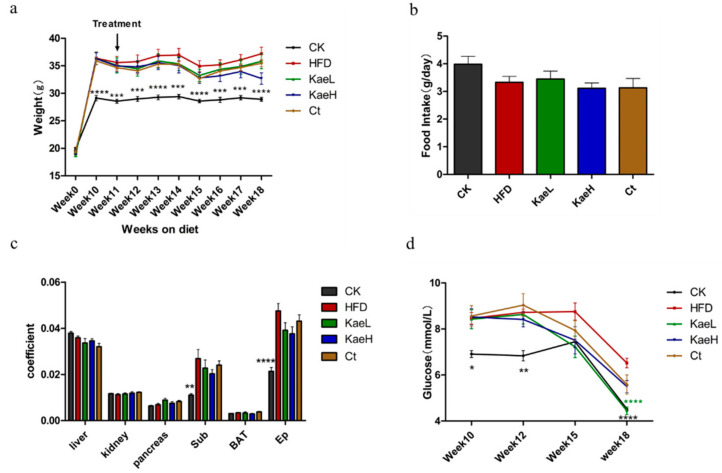
Effects of *C. tinctoria* and kaempferol on mouse physiology and glucose. (**a**) Weekly body weight of mice. (**b**) Mean daily food intake. (**c**) Organ coefficient. (**d**) Fasting blood glucose. Sub: subcutaneous fat, BAT: brown adipose tissue, Ep: epididymal fat, CK: control group, HFD: high-fat diet group, Ct: HFD+water extract of *C. tinctoria* group, KaeL: HFD+kaempferol low group, KaeH: HFD+kaempferol high group. Value = mean ± SD (n = 8). * *p* < 0.05 vs. HFD, ** *p* < 0.01 vs. HFD, *** *p* < 0.001 vs. HFD, **** *p* < 0.0001 vs. HFD.

To explore the effects of *C. tinctoria* and kaempferol on hyperglycemia in obese mice, an obese mouse model was successfully established by feeding HFD. After 10 weeks, the body weight and fasting blood glucose of the HFD (before treatment with *C. tinctoria* and kaempferol) were significantly elevated compared to those of the control group. During the administration of *C. tinctoria*, the weight of mice in the Ct group was lower than that in HFD group, although the difference was not significant (Figure 1a). Treatment with kaempferol also reduced the body weight gain; especially, the KaeH was more effective in ameliorating the increase in body weight. During the 18-week feeding, no significant difference in food intake was observed between the treatment group and the HFD group (Figure 1b). Similar results were observed in organ coefficients (Figure 1c). Furthermore, the fasting blood glucose of *C. tinctoria* group appeared to decrease at the 15th week and kaempferol groups reached a significant level at the 18th week (Figure 1d) (*p* < 0.0001). These results show that *C. tinctoria* and kaempferol effectively improved the obesity of hyperglycemic mice induced by HFD without affecting the food intake of DIO mice.

### 3.3. C. tinctoria and Kaempferol Ameliorated Glucose Metabolism in DIO Mice

**Figure 2:**
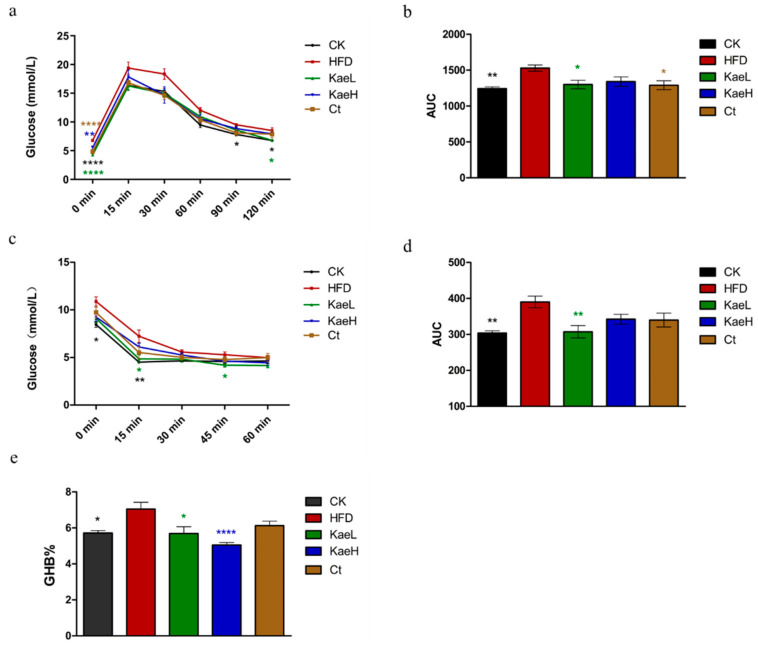
Effects of *C. tinctoria* and kaempferol on glucose metabolism in DIO mice. (**a**) Glucose tolerance test (GTT). (**b**) The area under the curve (AUC) of GTT. (**c**) Insulin tolerance test (ITT). (**d**) The AUC of ITT. (**e**) Hemoglobin a1c (HbA1c) concentrations of whole blood. GHB: glycated hemoglobin, CK: control group, HFD: high-fat diet group, Ct: HFD+water extract of *C. tinctoria* group, KaeL: HFD+kaempferol low group, KaeH: HFD+kaempferol high group. Value = mean ± SD (n = 8). * *p* < 0.05 vs. HFD, ** *p* < 0.01 vs. HFD, **** *p* < 0.0001 vs. HFD.

In order to determine the hypoglycemic effects of *C. tinctoria* and kaempferol, GTTs and ITTs were performed at the 17th week and 16th week, respectively. The 16 h fasting blood glucose levels of the HFD group were significantly elevated compared to the CK group (*p* < 0.0001), and treatment with *C. tinctoria* and kaempferol significantly mitigated HFD-induced blood glucose elevation (Figure 2a,b) (*p* < 0.05). The results of GTTs show that *C. tinctoria* and kaempferol could effectively improve glucose tolerance of DIO mice. Significant differences were observed in insulin tolerance for the 4 h fasting blood glucose levels (Figure 2c,d). Insulin sensitivities of the HFD group were impaired compared to the CK group, but kaempferol significantly reversed the insulin resistance induced by high-fat diet. Based on the above results, *C. tinctoria* and kaempferol could effectively lower serum glucose and improve glucose intolerance and insulin resistance in DIO mice.

If HbA1c > 9% indicates that patients have persistent hyperglycemia, complications such as diabetic nephropathy, arteriosclerosis, and cataract will occur [21,22]. As shown in Figure 2e, kaempferol groups showed significantly lower concentrations of HbAc1 compared with HFD groups in DIO mice.

### 3.4. C. tinctoria and Kaempferol Improve Obesity without Effect on the Liver and Kidney in DIO Mice

**Figure 3:**
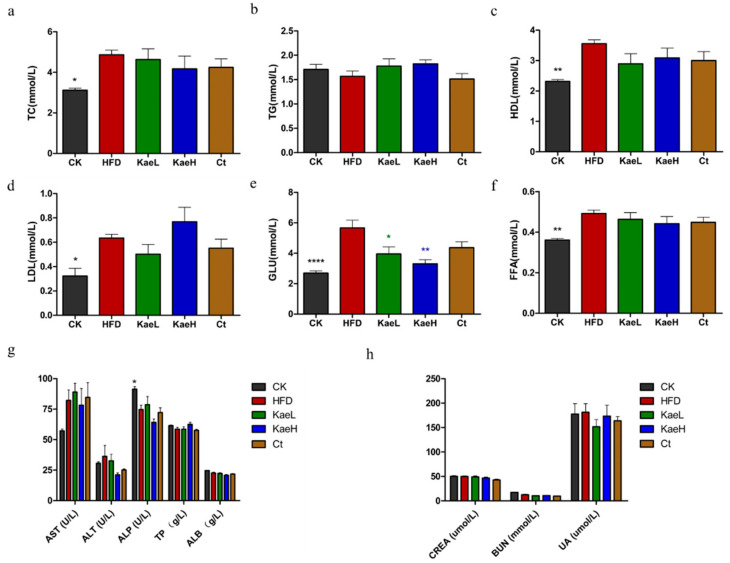
Effects of *C. tinctoria* and kaempferol on biochemical parameters of serum. (**a**) TC. (**b**) TG. (**c**) HDL. (**d**) LDL. (**e**) GLU. (**f**) FFA. (**g**) Liver function parameters. (**h**) Kidney function parameters. TC: total cholesterol, TG: triglyceride, HDL: high-density lipoprotein, LDL: low-density lipoprotein, GLU: glucose, FFA: free fatty acid, AST: aspartate transaminase, ALT: alanine aminotransferase, ALP: alkaline phosphatase, TP: total protein, ALB: albumin, CREA: creatinine, BUN: blood urea nitrogen, UA: uric acid, CK: control group, HFD: high-fat diet group, Ct: HFD+water extract of *C. tinctoria* group, KaeL: HFD+kaempferol low group, KaeH: HFD+kaempferol high group. Value = mean ± SD (n = 8). * *p* < 0.05 vs. HFD, ** *p* < 0.01 vs. HFD, **** *p* < 0.0001 vs. HFD.

The concentrations of serum TC, TG, HDL, LDL, and FFA in all groups were determined at 8 weeks of administration (Figure 3a–d,f). TC, HDL, and FFA levels were reversed in the HFD group after treatment with *C. tinctoria* and kaempferol, but no significant difference was observed. Moreover, kaempferol significantly decreased serum glucose levels in DIO mice compared to the HFD group (Figure 3e) (*p* < 0.01). In addition, no significant changes in liver or kidney function indicators were observed in the administration groups compared to the HFD groups (Figure 3g,h). These results indicate that *C. tinctoria* and kaempferol were capable of mitigating obesity progress, and had no effect on liver and kidney function parameters.

### 3.5. Effect of C. tinctoria and Kaempferol on Gut Microbe in DIO Mice

**Figure 4:**
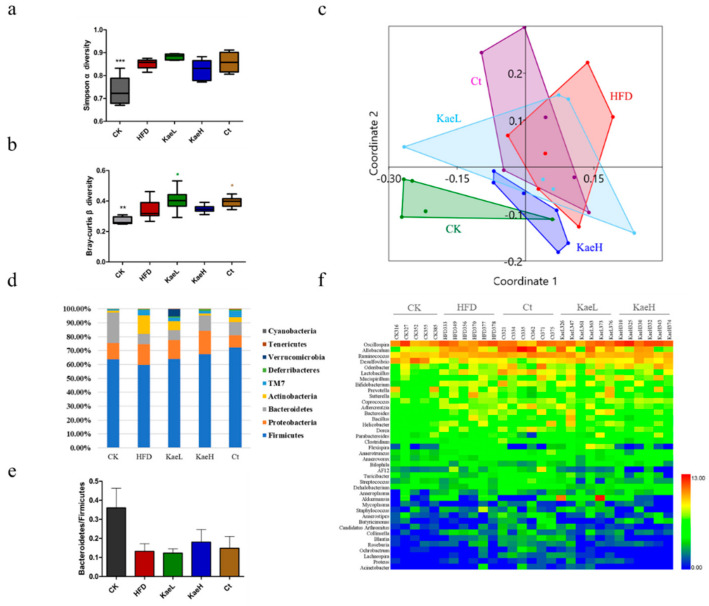
Effect of *C. tinctoria* and kaempferol on gut microbe. (**a**) Simpson α diversity index among groups. (**b**) Bray-Curtis β diversity index among groups. (**c**) The NMDS graph shows the two-dimensional plane distribution of each group of animals (that is, the degree of difference between groups characterized by β diversity). (**d**) Microbial community analysis at the phylum level in mice. (**e**) The alteration in the Bacteroidetes to Firmicutes ratio in mice. (**f**) Heat map analysis of microbial community at the genus level in mice. CK: control group, HFD: high-fat diet group, Ct: HFD+water extract of *C. tinctoria* group, KaeL: HFD+kaempferol low group, KaeH: HFD+kaempferol high group. Value = mean ± SD (n = 8). * *p* < 0.05 vs. HFD, ** *p* < 0.01 vs. HFD, *** *p* < 0.001 vs. HFD, **** *p* < 0.0001 vs. HFD.

To evaluate the impact of *C. tinctoria* and kaempferol treatment on gut microbiota diversity, the α and β diversity of all groups were measured (Figure 4a,b). The α diversity had no significant difference, while the β diversity was significantly elevated after treatment with *C. tinctoria* and kaempferol compared to the HFD groups (*p* < 0.05). The analysis of nonmetric multidimensional scaling analysis (NMDS) showed that there was a significant difference in gut microbial composition between high-fat diet-induced obese hyperglycemic mice and normal diet mice (*p* < 0.01, Figure 4c). Administration of *C. tinctoria* and kaempferol had significant effects on the composition of intestinal flora in DIO mice and enabled the composition of HFD mice to migrate to that of CK mice.

As shown in Figure 4d, *Firmicutes*, *proteus*, *Bacteroidetes*, and *actinomycetes* were important components of the gut microbe, accounting for more than 90%, and there were also some *TM7*, *desferribacilli*, *verruca*, *soft wall bacteria*, and *cyanobacteria* in all groups. Compared with the CK group, *Bacteroidetes* in the HFD group decreased. After treatment with *C. tinctoria* and kaempferol, *Bacteroidetes* increased. *Actinomycetes* increased from 1.34% to 13.51% compared with CK to HFD. *C. tinctoria* and kaempferol treatment reduced *Actinomycetes* in high-fat diet mice. Similar improvements were observed for *Bacteroidetes/Firmicutes* (Figure 4e).

Heat map cluster analysis of gut microbiota was performed, and the results are illustrated in Figure 4f. As expected, there was a significant difference in intestinal flora between HFD and CK, represented as *Dehalobacterium* of the *phylum Firmicutes* decreasing significantly (*p* < 0.01), *Desulfovibrio* of the *phylum Proteus* decreasing significantly (*p* < 0.01), and *collinella* of the *phylum actinobacteriaceae* increasing significantly (*p* < 0.01). These changes were significantly reversed in Ct, KaeL, and KaeH groups (*p* < 0.01).

**Figure 5:**
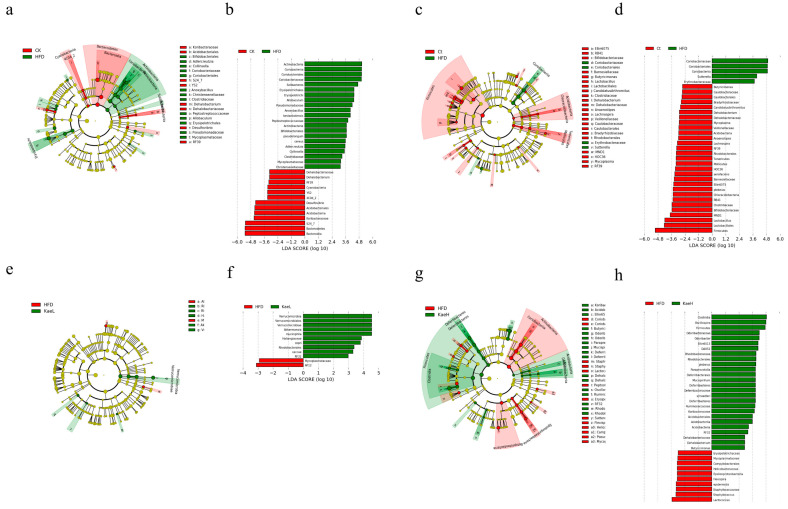
LEfSe cluster analysis of the structure of gut microbiome. Linear discriminant analysis effect size (LEfse) results are represented by circular cladograms (**a**,**c**,**e**,**g**). Differentially expressed taxa are highlighted by colored and shaded circles. The diameter of the circle is related to abundance of taxa. (**a**) CK vs. HFD; (**c**) Ct vs. HFD; (**e**) KaeL vs. HFD; (**g**) KaeH vs. HFD. Linear discriminant analysis (LDA) results are shown in figure (**b**,**d**,**f**,**h**). The length of the bar represents LDA score, and the colors of the bar represent the respective groups. The threshold on the logarithmic LDA score was set to 2.0. The taxon of bacteria with significant changes (*p* < 0.05) in relative abundance is written alongside the horizontal lines. (**b**) Red represents CK, green represents HFD; (**d**) red represents Ct, green represents HFD; (**f**) red represents HFD, green represents KaeL; (**h**) red represents HFD, green represents KaeH. CK: control group, HFD: high-fat diet group, Ct: HFD+water extract of *C. tinctoria* group, KaeL: HFD+kaempferol low group, KaeH: HFD+kaempferol high group.

The results of LEfSe cluster analysis were consistent with those of NMDS analysis. There was a significant decrease in *dehalobacterium* (*p* < 0.05), *Desulfovibrio* (*p* < 0.05), s24-7 (*muribaculaceae*), and *Collins* (*coriobacteriaeae*) of *actinobacteria* (*collinsella*). The content of *Bifidobacterium pseudolongum, allobaculum*, and *Bacillus cereus* increased significantly (*p* < 0.05) compared with CK (Figure 5a,b). After treatment with *C. tinctoria*, the number of *Vibrio butyricimonas* (*p* < 0.01), *dehalobacterium*, *anaerostipes*, *lachnospira*, and *Mycoplasma* increased significantly (*p* < 0.05) compared with the HFD group (Figure 5c,d), while *muciniphila* of *akkermansia* and *caccae* of *bacteroidea* decreased significantly (*p* < 0.05) in KaeL (Figure 5e,f). *Oscillospira* (*p* < 0.05), *schaedleri* species (*p* < 0.05), *dehalobacterium* (*p* < 0.05), and *Vibrio butyricimonas* (*p* < 0.01) of *Firmicutes* increased significantly and *Proteus flexispira* and *sutterella* decreased significantly (*p* < 0.05) in KaeH (Figure 5g,h). Therefore, *C. tinctoria* and kaempferol treatment ameliorated the whole gut microbe in DIO mice.

## 4. Discussion

*C. tinctoria* has been used in tea and food for many years [13]. The beneficial effects of *C. tinctoria* mainly consist of anti-inflammatory and antioxidant effects, which are attributed to its flavonoids [23,24,25]. In addition, polysaccharides, phenylpropanoids, polyacetylenes, and other substances in *C. tinctoria* also have certain pharmacological activities [26,27,28]. However, these components have not attracted enough attention at present. Here, we verified that the extracts of *C. tinctoria* and its flavonoid kaempferol could reduce blood glucose and improve insulin sensitivity partly through regulating gut microbiota.

In this study, fasting blood glucose levels significantly decreased with kaempferol treatment in high-fat diet-induced obese mice, but there was no significant difference in the Ct group. It has been observed in cell and animal experiments that kaempferol can improve obesity by inhibiting adipogenesis [29,30].

In GTT and ITT experiments, both *C. tinctoria* and kaempferol significantly ameliorated glucose tolerance and insulin sensitivity compared with the HFD group. It has been reported that the extracts of *C. tinctoria* could significantly reduce blood glucose of diabetic mice [31]. Similar studies also showed that daily administration of kaempferol significantly decreased oral glucose tolerance, intraperitoneal insulin tolerance, and serum lipids [32].

In addition, *C. tinctoria* and kaempferol reduced TC, HDL, FFA, and glycosylated hemoglobin, and improved glucose metabolism and lipid metabolism in DIO mice. This is consistent with previous research results [33].

We also observed that the treatment of *C. tinctoria* and kaempferol had a significant effect on intestinal flora. Most obviously, the genus *dehalobacterium* of *Firmicutes* and *Desulfovibrio* of *Proteus* decreased significantly (*p* < 0.01), and *collinella* of *actinomycetes* increased significantly (*p* < 0.01) in HFD compared with CK, while *C. tinctoria* and kaempferol could significantly reverse the changes of these flora (*p* < 0.01). Previous research has shown that there is a correlation between *collinella* and insulin. The increase in *Desulfovibrio* can improve glucose metabolism, and the decrease in *dehalobacterium* will lead to premature aging, which is consistent with the results of *collinella* and *Desulfovibrio* regulating glucose metabolism in the literature [34,35,36]. The treatment of *C. tinctoria* and kaempferol can increase short-chain fatty acid-producing bacteria, such as *butyrimionas* (*Vibrio butyricum*), which is consistent with reports that short-chain fatty acids and other bioactive components can target intestines, liver, and other organs so as to improve intestinal health, control blood glucose, and regulate insulin resistance [37]. It was initially reported that obesity was associated with a lower proportion of *Bacteroides* than *Firmicutes*. However, this view was challenged by later studies [38].

## 5. Conclusions

In conclusion, *C. tinctoria* and its flavonoid kaempferol have protective effects on glucose metabolism disorder and changes in gut microbe in diet-induced obese mice. The alleviating effect may be related to the increase in bacteria regulating glucose metabolism and producing short-chain fatty acids.

## Figures and Tables

**Table 1:** Components of *C. tinctoria*.

Component	Retention Time (min)	Peak Area (mV·min)	Adduct Type
Eriodictyol	4.57	4.42 × 10^7^	[M+H]+
Kaempferol	5.04	8.54 × 10^6^	[M+H]+
Glycine-Betaine	0.96	7.23 × 10^6^	[M+H]+
N-Fructosyl isoleucine	1.78	7.02 × 10^6^	[M+H]+
Taxifolin	4.62	2.94 × 10^6^	[M-H]-
Luteolin	5.83	2.63 × 10^6^	[M+H]+
Anileridine	6.32	2.04 × 10^6^	[M+H]+
3,5-Dicaffeoylquininic acid	4.55	1.66 × 10^6^	[M-H]-
4-Methoxycinnamic acid	6.03	1.66 × 10^6^	[M-H]-
Caffeoyl quinic acid	3.37	1.46 × 10^6^	[M-H]-
Coumaric acid	6.27	1.46 × 10^6^	[M+H-H_2_O]+
Quercetin-3-O-glucoside	4.46	1.45 × 10^6^	[M+H]+
Eriodictyol-7-O-glucoside	3.90	1.32 × 10^6^	[M+H]+
N,N-Dimethyldodecylamine N-oxide	6.62	1.29 × 10^6^	[M+H]+
Luteolin-4’-O-glucoside	4.90	1.24 × 10^6^	[M+H]+
Luteolin-7-O-glucoside	4.33	1.03 × 10^6^	[M+H]+
isookanin-7-O-glucoside	4.25	8.47 × 10^5^	[M-H]-
Naringenin	6.37	7.43 × 10^5^	[M+H]+
Quercetin-3-O-glucoside; PlaSMA ID-1264	4.27	3.39 × 10^5^	[M-H]-
Caffeic acid	4.06	3.28 × 10^5^	[M+H]+
Hyperoside	4.93	2.70 × 10^5^	[M+H]+
Naringenin-7-O-glucoside	5.16	2.66 × 10^5^	[M+H]+

## Data Availability

Data are available from the corresponding author (hexiaoyun@cau.edu.cn) upon reasonable request.

## References

[1] Said M.M., Sandra S., Jaime L., Ignacio B. (2018). Systems and WBANs for Controlling Obesity. J. Healthc. Eng..

[2] Polsky S., Ellis S.L. (2015). Obesity, insulin resistance, and type 1 diabetes mellitus. Curr. Opin. Endocrinol. Diabetes Obes..

[3] Blüher M. (2019). Obesity: Global epidemiology and pathogenesis. Nat. Rev. Endocrinol..

[4] Malone J.I., Hansen B.C. (2019). Does obesity cause type 2 diabetes mellitus (T2DM)? Or is it the opposite?. Pediatr. Diabetes.

[5] Pulgaron E.R., Delamater A.M. (2014). Obesity and Type 2 Diabetes in Children: Epidemiology and Treatment. Curr. Diabetes Rep..

[6] Leitner D.R., Frühbeck G., Yumuk V., Schindler K., Micic D., Woodward E., Toplak H. (2017). Obesity and Type 2 Diabetes: Two Diseases with a Need for Combined Treatment Strategies—EASO Can Lead the Way. Obes. Facts.

[7] Simonson M., Boirie Y., Guillet C. (2020). Protein, amino acids and obesity treatment. Rev. Endocr. Metab. Disord..

[8] Caballero B. (2019). Humans against Obesity: Who Will Win?. Adv. Nutr..

[9] Chao A.M., Quigley K.M., Wadden T.A. (2021). Dietary interventions for obesity: Clinical and mechanistic findings. J. Clin. Investig..

[10] Shen J., Hu M., Tan W., Ding J., Xiao P. (2020). Traditional uses, phytochemistry, pharmacology, and toxicology of *Coreopsis tinctoria* Nutt.: A review. J. Ethnopharmacol..

[11] Jiang B., Le L., Wan W., Zhai W., Hu K., Xu L., Xiao P. (2015). The Flower Tea *Coreopsis tinctoria* Increases Insulin Sensitivity and Regulates Hepatic Metabolism in Rats Fed a High-Fat Diet. Endocrinology.

[12] Guo Y., Wang D., Cheng X., Chen Y., Duan M., Wei L. (2016). The Preliminary Screening of Antihypertension Activity Effective Parts for Hotan *Coreopsis tinctoria* Nutt. J. Yunnan Univ. Tradit. Chin. Med..

[13] Li Y., Yang P., Gao B., Sun J., Lu W., Liu J., Chen P., Zhang L.Y. (2019). Chemical compositions of chrysanthemum teas and their anti-inflammatory and antioxidant properties. Food Chem..

[14] Ren Z., Li Y., Liu J., Li H., Li A., Hong L., Cui G., Sun R., Wulasihan M., Sun J. (2018). *Coreopsis tinctoria* Modulates Lipid Metabolism by Decreasing Low-Density Lipoprotein and Improving Gut Microbiota. Cell. Physiol. Bio-Chem..

[15] Jiang B., Lv Q., Wan W., Le L., Xu L., Hu K., Xiao P. (2018). Transcriptome analysis reveals the mechanism of the effect of flower tea *Coreopsis tinctoria* on hepatic insulin resistance. Food Funct..

[16] Guo Y., Ran Z., Zhang Y., Song Z., Wang L., Yao L., Zhang M., Xin J., Mao X. (2020). Marein ameliorates diabetic nephropathy by inhibiting renal sodium glucose transporter 2 and activating the AMPK signaling pathway in db/db mice and high glucose-treated HK-2 cells. Biomed. Pharmacother..

[17] Begmatov N., Li J., Bobakulov K., Numonov S., Aisa H.A. (2018). The chemical components of *Coreopsis tinctoria* Nutt. and their antioxidant, antidiabetic and antibacterial activities. Nat. Prod. Res..

[18] Li Y., Chen X., Xue J., Liu J., Chen X. (2014). Muhuyati Wulasihan Flavonoids furom *Coreopsis tinctoria* adjust lipid metabolism in hyperlipidemia animals by down-regulating adipose differentiation-related protein. Lipids Health Dis..

[19] Lan S., Lin J., Zheng N. (2014). Evaluation of the antioxidant activity of *Coreopsis tinctoria* nuff. and optimisation of isolation by response surface methodology. Acta Pharm..

[20] Imran M., Salehi B., Sharifi-Rad J., Gondal T.A., Saeed F., Imran A., Shahbaz M., Fokou P.V.T., Arshad M.U., Khan H. (2019). Kaempferol: A Key Emphasis to Its Anticancer Potential. Molecules.

[21] Campbell L., Pepper T., Shipman K. (2019). HbA1c: A review of non-glycaemic variables. J. Clin. Pathol..

[22] Claesson R., Ignell C., Shaat N., Berntorp K. (2017). HbA1c as a predictor of diabetes after gestational diabetes mellitus. Prim. Care Diabetes.

[23] Zhang Y., Shi S., Zhao M., Chai X., Tu P. (2013). Coreosides A–D, C14-polyacetylene glycosides from the capitula of *Coreopsis tinctoria* and its anti-inflammatory activity against COX-2. Fitoterapia.

[24] Zhang W., Sun Q., Zheng W., Zhang Y., Du J., Dong C., Tao N. (2019). Structural characterization of a polysaccharide from *Coreopsis tinctoria* Nutt. and its function to modify myeloid derived suppressor cells. Int. J. Biol. Macromol..

[25] Li Y., Huang C., Fu W., Zhang H., Lao Y., Zhou H., Tan H., Xu H. (2020). Screening of the active fractions from the *Coreopsis tinctoria* Nutt. Flower on diabetic endothelial protection and determination of the underlying mechanism. J. Ethnopharmacol..

[26] Hoppe W., Schramm H.J., Sturm M., Hunsmann N., Gaßmann J. (1989). Accumulation of Unusual Phenylpropanoids in Transformed and Non-Transformed Root Cultures of *Coreopsis tinctoria*. Z. Nat. C.

[27] Liu Y., Du D., Liang Y., Xin G., Huang B., Huang W. (2015). Novel polyacetylenes from *Coreopsis tinctoria* Nutt. J. Asian Nat. Prod. Res..

[28] Jing S., Chai W., Guo G., Zhang X., Dai J., Yan L. (2016). Comparison of antioxidant and antiproliferative activity between Kunlun Chrysanthemum flowers polysaccharides (KCCP) and fraction PII separated by column chromatography. J. Chromatogr. B. Anal. Technol. Biomed. Life Sci..

[29] Torres-Villarreal D., Camacho A., Castro H., Ortiz-Lopez R., de la Garza A.L. (2019). Anti-obesity effects of kaempferol by inhibiting adipogenesis and increasing lipolysis in 3T3-L1 cells. J. Physiol. Biochem..

[30] Wang T., Wu Q., Zhao T. (2020). Preventive Effects of Kaempferol on High-Fat Diet-Induced Obesity Complications in C57BL/6 Mice. BioMed Res. Int..

[31] Dias T., Bronze M.R., Houghton P.J., Mota-Filipe H., Paulo A. (2010). The flavonoid-rich fraction of *Coreopsis tinctoria* promotes glucose tolerance regain through pancreatic function recovery in streptozotocin-induced glucose-intolerant rats. J. Ethnopharmacol..

[32] Chen Y., Zhang C., Jin M., Qin N., Qiao W., Yue X., Duan H., Niu W. (2016). Flavonoid derivative exerts an antidiabetic effect via AMPK activation in diet-induced obesity mice. Nat. Prod. Res..

[33] Cai W., Yu L., Zhang Y., Feng L., Kong S., Tan H., Xu H., Huang C. (2016). Extracts of *Coreopsis tinctoria* Nutt. Flower Exhibit Antidiabetic Effects via the Inhibition of α-Glucosidase Activity. J. Diabetes Res..

[34] Bárcena C., Valdés-Mas R., Mayoral P., Garabaya C., Durand S., Rodrígue F., Fernández-García M.T., Salazar N., Nogacka A.M., Garatachea N. (2019). Healthspan and lifespan extension by fecal microbiota transplantation into progeroid mice. Nat. Med..

[35] Gomez-Arango L.F., Barrett H.L., Wilkinson S.A., Callaway L.K., McIntyre H.D., Morrison M., Nitert M.D. (2018). Low dietary fiber intake increases Collinsella abundance in the gut microbiota of overweight and obese pregnant women. Gut Microbes.

[36] Pichette J., Fynn-Sackey N., Gagnon J. (2017). Hydrogen Sulfide and Sulfate Prebiotic Stimulates the Secretion of GLP-1 and Improves Glycemia in Male Mice. Endocrinology.

[37] Nie Q., Chen H., Hu J., Fan S., Nie S. (2019). Dietary compounds and traditional Chinese medicine ameliorate type 2 diabetes by modulating gut microbiota. Crit. Rev. Food Sci. Nutr..

[38] Liu R., Hong J., Xu X., Feng Q., Zhang D., Gu Y., Shi J., Zhao S., Liu W., Wang X. (2017). Gut microbiome and serum metabolome alterations in obesity and after weight-loss intervention. Nat. Med..

